# Capacity and kinetics of light-induced cytochrome oxidation in intact cells of photosynthetic bacteria

**DOI:** 10.1038/s41598-022-18399-y

**Published:** 2022-08-22

**Authors:** Mariann Kis, James L. Smart, Péter Maróti

**Affiliations:** 1 Department of Medical Physics and Informatics, University of Szeged, Rerrich Béla tér 1, 6720 Szeged, Hungary; 2 Balaton Limnological Research Institute, 8237 Tihany, Hungary; 3 Department of Biological Sciences, University of Tennessee at Martin, Martin, TN 38238 USA

**Keywords:** Bioenergetics, Membrane biophysics, Molecular biophysics

## Abstract

Light-induced oxidation of the reaction center dimer and periplasmic cytochromes was detected by fast kinetic difference absorption changes in intact cells of wild type and cytochrome mutants (*cycA*, *cytC4* and *pufC*) of *Rubrivivax* *gelatinosus* and *Rhodobacter* *sphaeroides*. Constant illumination from a laser diode or trains of saturating flashes enabled the kinetic separation of acceptor and donor redox processes, and the electron contribution from the cyt *bc*_1_ complex via periplasmic cytochromes. Under continuous excitation, concentrations of oxidized cytochromes increased in three phases where light intensity, electron transfer rate and the number of reduced cytochromes were the rate liming steps, respectively. By choosing suitable flash timing, gradual steps of cytochrome oxidation in whole cells were observed; each successive flash resulted in a smaller, damped oxidation. We attribute this damping to lowered availability of reduced cytochromes resulting from both exchange (unbinding/binding) of the cytochromes and electron transfer at the reaction center interface since a similar effect is observed upon deletion of genes encoding periplasmic cytochromes. In addition, we present a simple model to calculate the damping effect; application of this method may contribute to understanding the function of the diverse range of c-type cytochromes in the electron transport chains of anaerobic phototrophic bacteria.

## Introduction

In purple non-sulphur bacteria, electron and proton transfer reactions convert light energy into biochemically amenable energy that covers the energy cost required for these organisms to live. The primary process of photosynthesis is initiated by the absorption of light via light-harvesting pigments followed by the transfer of the electronic excitation energy to the special pair, P, of the reaction center (RC) protein^1–3^. The excitation of the special pair (P*) leads to charge separation (P^+^Q_A_^─^) by oxidation of P and passage of the electron to the primary quinone (Q_A_) of the acceptor complex via bacteriochlorophyll monomer and bacteriopheophytin pigments. This is subsequently followed by electron transfer to the secondary quinone Q_B_. After repetition of the process, Q_B_ is fully reduced by two electrons and two protons (Q_B_H_2_), unbinds from the RC and carries the electrons and protons through the membrane^4,5^. On the donor side of the RC, P^+^ is re-reduced by a periplasmic reduced cytochrome so that the charge separation process can start anew^6^. Soluble periplasmic electron carrier proteins transport electrons to the RC after each turnover and are re-reduced in turn by a quinol molecule via the cytochrome *bc*_1_ complex. This cyclic electron transfer occurring between these different electron carriers is coupled to the uptake and translocation of protons across the cytoplasmic membrane, creating a protonmotive force that drives ATP synthesis.

The kinetics and stoichiometry of electron transfer in the quinone acceptor complex are well established and highly similar in different strains of purple bacteria^4,7^. Q_A_ is reduced within 100 ps via charge separation (PQ_A_ → P^+^Q_A_^─^) and re-oxidized by Q_B_ in 100 μs via interquinone electron transfer (Q_A_^─^Q_B_ → Q_A_Q_B_^─^). While Q_A_ (ubiquinone in *Rba.* *sphaeroides* but menaquinone in *Rvx.* *gelatinosus* and *Bl.* *viridis*) performs one-electron chemistry, Q_B_ (ubiquinone in all cases) is reduced fully by two electrons (and two protons). Thus, at most, three flashes could be absorbed by the RC without export of ubiquinol, since the first two flashes would result in a two-electron reduction of Q_B_. If a third flash were to arrive before exchange of Q_B_ with the membrane ubiquinone pool, this would result in the reduction of Q_A_ and a "closed" RC that is unable to absorb additional light energy. Once exchange of Q_B_ and subsequent oxidation of Q_A_ is achieved, the RC would be “open” and available to absorb photons from subsequent flashes. These electron transfers are much more rapid than the time scale for exchange of the Q_B_ site with the quinone pool, estimated in *Rba.* *sphaeroides* to be on the order of a millisecond^8^.

The donor side, however, is more tolerant of possible donors in different strains^9,10^. Many purple bacteria (e.g., *Rvx.* *gelatinosus*) have to the RC tightly bound tetraheme cytochrome that reduces the special pair after photo-induced electron transfer^11,12^. Additionally, several water-soluble electron carrier proteins are present in the periplasmic space of *Rvx.* *gelatinosus*. Four of these proteins (high potential iron-sulfur protein (HiPIP), high potential cytochrome c_8_, low potential cytochrome c_8_ and possibly cytochrome c_4_), have been shown to function as electron donors to the RC-bound cytochrome^13,14^. RCs of some purple bacteria (e.g., *Rba.* *sphaeroides*) do not have a bound cytochrome c subunit. Instead, they depend on a soluble cytochrome *c*_2_ or another soluble periplasmic electron transfer proteins like cyt c_4_, cyt *c*_8_ or cyt *c*_y_^15,16^. These soluble cytochromes play an essential role in photosynthetic energy metabolism of wild type cells since cyt *c*_2_ is required for photosynthetic growth of wild type cells^17,18^.

The cytochrome c_2_–RC interface of *Rba* *sphaeroides* was probed extensively by rapid kinetic measurements of electron transfer (ET) in a mixture of isolated RC and *c*_2_ cytochromes^18,19^. Following a single flash that photo-oxidizes the RC, there is a fast, ∼1 μs, transfer of an electron from cytochrome *c*_2_, ascribed to a proximal cytochrome ‘pre-bound’ to its site on the RC before the flash. A slower 50–200 μs phase in such kinetic assays likely reflects a series of processes that include the electrostatically-guided approach of the distal donor cytochrome arriving from the bulk phase forming an initial encounter complex. However, for intact cells, these kinetic measurements and the classification of these cytochromes as proximal or distal has not yet been confirmed.

The tetraheme subunit in *Bl.* *viridis* has alternatively two low potential (−60 mV and + 20 mV) and two high potential (+ 310 mV and + 380 mV) hemes to direct the flow of electron transfer from a periplasmic soluble electron donor to the oxidized special pair P^+^. The structure of the tetraheme subunit with a periplasmic cytochrome donor from *T.* *tepidum* was co-crystallized recently by Kawakami and coworkers^20^. In this study, the interaction surface between the tetraheme subunit and the periplasmic donor is shown to be mostly hydrophobic, which explains the variety of periplasmic cytochrome donors tolerated by the tetraheme subunit. Based on the mutational replacements of charged amino acid residues distributed on the surface of the subunit, Osyczka et al. have shown that the low potential heme located at the most distant position from the special pair is a direct electron acceptor from the soluble electron carrier, which suggests that all four hemes are involved in the electron transfer to the special pair^21^. However, direct evidence for the interaction mechanisms between the hemes and the soluble electron donor is not yet available. From calculated interheme effective electronic couplings, Burggraf & Koslowski concluded that at most the two heme molecules closest to P participate in a fast re-reduction of the oxidized dimer, whereas the remaining hemes are likely not necessary but useful in different functions, such as intermediate electron storage^22^.

Kinetic studies revealed that heme closest to P transfers an electron to the dimer in 100–200 ns depending on states of the heme groups^23^ which is then re-reduced on a time scale of 2 μs by an electron transfer involving the set of hemes^24^. Chen et al. have shown that the transfer rates of the inter-heme electron flow are particularly sensitive to changes in the redox potential of the hemes involved^25^. To our knowledge, direct evidence for the functional role of these hemes is still missing.

The arrangement of the hemes according to their midpoint redox potential reveals a unique feature of the subunit: it is able to move electrons uphill against the redox potential gradient. The distal heme of the tetraheme cytochrome has a much lower redox potential (−60 mV) than donor, cyt c_2_ (+ 345 mV), yet donates electron within 60 μs to the P^+^ state^26^. The tetraheme subunit can overcome this energy barrier; likely from the overall downhill thermodynamics of the electron transfer from cyt c_2_ to the RC. Additionally, the tetraheme cytochrome subunit attached firmly to the RC makes the electron transfer essentially independent of diffusion and unbinding/binding (exchange) of the periplasmic electron donor carrier. These features serve the structural and functional diversity of cytochromes in anoxygenic photosynthetic purple bacteria to survive in their challenging environments.

By measurement of time-resolved light-induced absorption changes specific to the special pair bacteriochlorophyll dimer and the cytochromes in whole cells of different strains and cytochrome mutants, we are seeking solution to important problems that remain unanswered. The observed kinetics of cytochrome oxidation evoked by either stationary or flash illumination involves a pairing of donor and acceptor side reactions, whose individual contributions have not yet been resolved^27,28^. By proper choice of illumination (a train of intensive flashes), conditions can be set where the turnover of the donor side will be the rate limiting step. This method can be used to measure the availability of reduced cytochromes and thus the capacity of the system, which has been always an essential and hard-to-answer question in intact bacteria. In the present study, we investigate how the availability of reduced cytochromes drops, or appears to be damped during illumination by successive flashes, and what factors control this damping effect. It will be shown that oxidation of the donor side decreases this damping effect, whether naturally (photooxidation by flash) or artificially (oxidation by potassium ferricyanide).

## Results

### (1) Absorption difference spectra of cytochromes in wild type and mutant bacteria

**Figure 1:**
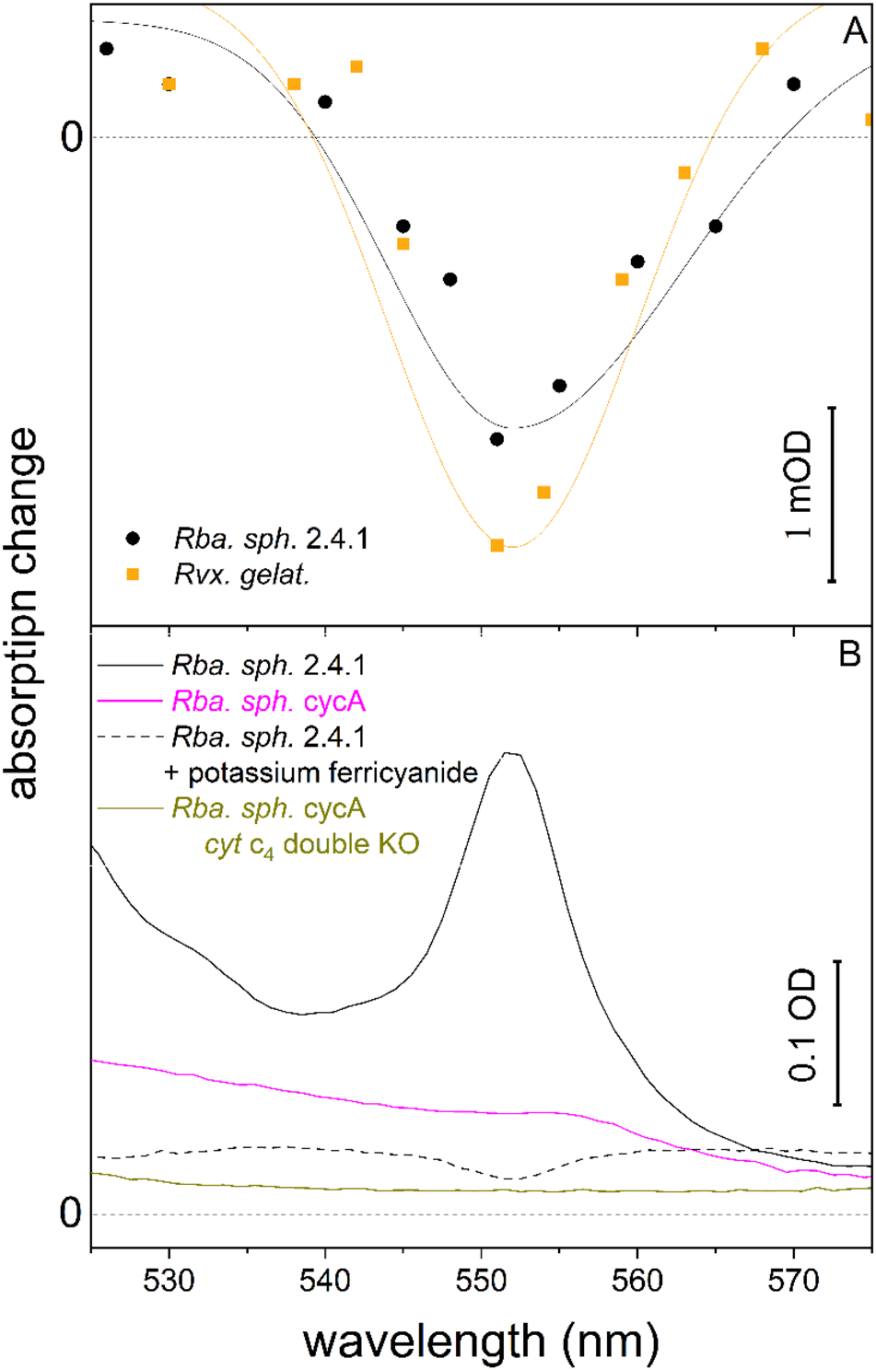
Spectra of absorption change caused by redox changes of cytochromes of intact cells (**A**) and chromatophores (**B**) of photosynthetic purple bacteria. (**A**) Spectra of flash-induced oxidation of cytochromes of whole cells of *Rba.* *sphaeroides* 2.4.1 and *Rvx.* *gelatinosus* using the cytochrome-less mutant *cycA* with ferrocene (100 μM) as a baseline. (**B**) Steady state reduced minus oxidized absorption spectra of chromatophores prepared from wild type *Rba.* *sphaeroides* 2.4.1 and cytochrome-less mutants *cycA* and *cycA* *cytC4* (double KO). The samples were reduced by 1 mM sodium ascorbate and oxidized by 800 μM potassium ferricyanide. The absorbance changes were measured at room temperature.

We measured the flash-induced oxidation of donor *c*-type periplasmic cytochromes in whole cells of wild-type and mutant strains of *Rba.* *sphaeroides* and *Rvx.* *gelatinosus* (Fig. 1A). This was accomplished by monitoring the loss of the characteristic spectrum of reduced cytochromes: the α-band centered at 551 nm, relative to two isosbestic points (around 540 and 570 nm). We eliminated the potential confounding spectral contributions of the quinone Q/Q^─^ by establishing a baseline using the *cycA* strain of *Rba.* *sphaeroides* in the presence of the fast electron donor to P^+^. Thus, this method is sensitive only to those cytochromes that are directly oxidized by P^+^. We used the absorption coefficient of 20 mM^−1^ cm^−1^^29^, it serves as a quantitative measure of the photo-oxidized cytochrome *c* in the cells ([cyt *c*] ≈ 0.1–1 μM).

Cytochromes can also be quantified by determination of difference spectra for chemically reduced (ascorbate) minus oxidized (ferricyanide) cytochromes in chromatophores, as shown in Fig. 1B. However, with this method, all soluble and membrane-bound *c*-type cytochromes contribute to the observed spectrum. Since there is uncontrolled loss of cytochromes during the preparation (sonication and ultracentrifugation) of the chromatophores, the comparison of these data with the flash-induced data is difficult. We note that wild-type *Rba.* *sphaeroides* exhibits the 551-nm absorbance band characteristic of *c*-type cytochromes. However, neither its single (*cycA*) nor its double (*cycA* *cytC4*) cytochrome-less mutants exhibit a detectable absorbance feature around 551 nm, suggesting that the *c*-type cytochrome production was prevented in these strains.

### (2) Cytochrome oxidation under continuous (laser diode) illumination

**Figure 2:**
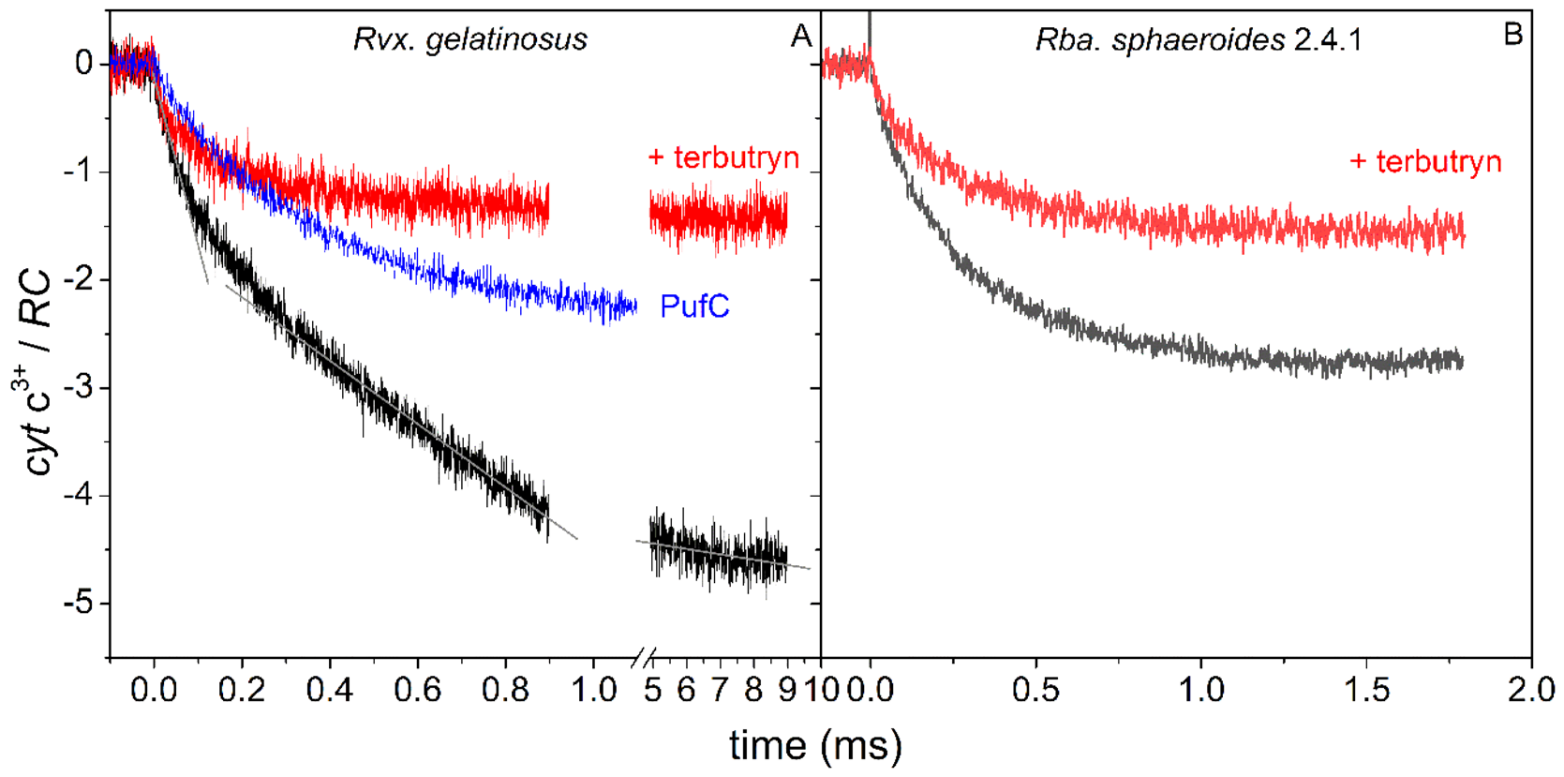
Kinetics of cytochrome *c* oxidation upon strong (2 W focussed) and continuous laser diode excitation (802 nm) in intact cells of *Rvx.* *gelatinosus* (**A**) and *Rba.* *sphaeroides* (**B**). The kinetic phases with descending rate constants (slopes of the straight lines) of 1.5·10^4^, 3.0·10^3^ and 50 cyt *c*^3+^/RC/s can be well distinguished in wild type *Rvx.* *gelatinosus* and are determined by different rate-limiting steps as (1) the intensity of the light (initial phase), (2) the rate of electron transfer (stationary phase) and (3) the size of the cytochrome pool (saturation). The absorption change upon oxidation of the cytochromes is negative (see Fig. 1A). The trace of the *PufC* mutant of *Rvx.* *gelatinosus* that lacks a cytochrome subunit but retains intact periplasmic cytochromes shows limited (partial) photooxidation compared to that of wild type. As reference, 100 μM terbutryne, an interquinone electron transfer inhibitor was added to the sample to block the turnover of the RC and to force the observation of only single cytochrome oxidation events.

The kinetics and capacity of oxidation of cytochrome *c* by the light-oxidized RC dimer (P^+^) can be determined in intact cells if the bacteria are exposed to excitation by a laser diode of appropriately high intensity. Upon focusing a laser with of 802 nm wavelength and 2 W power on a 1 × 1 mm^2^ spot of a culture of whole cells of *Rvx.* *gelatinosus*, the monotonous increase of the amount of the observed oxidized cytochrome *c* occurs in three distinct kinetic phases (Fig. 2A). The initial rise follows the primary photochemical charge separation PQ_A_ → P^+^Q_A_^─^ and P^+^ is immediately re-reduced by one of the hemes in the bound tetraheme cytochrome subunit. Thus, the rate constant of this phase (the slope of the straight line, 1.5·10^4^ cyt c^3+^/RC/s) is controlled by the light intensity.

The second linear segment describes the stationary accumulation of the oxidized cytochromes whose rate constant (3.0·10^3^ cyt c^3+^/RC/s) is limited not by the light intensity but by the rate constant of the cyclic electron transfer around the RC. The rate of the cyclic electron transfer depends also on the light intensity (see Fig. S2) but this dependence is weaker than in the case of the charge separation (the initial phase, described above). Therefore, a clear breakpoint separates these two straight lines since they have different slopes. The linearity of this phase remains as long as the availability of reduced cytochromes on the donor side is assured (for about 1 ms in our case), during which time cyclic electron flow occurs with a constant rate.

However, the pool of reduced cytochrome *c* starts to be exhausted later (about 10 ms); the rate constant decreases (50 cyt c^3+^/RC/s), and the kinetics become saturated. The saturation level can be substantially larger than 3 cyt c_2_^3+^/RC indicating the existence of a large pool of reduced cytochromes and the possibility of several turnovers of the RC. In strains where the pool size is significantly smaller, the saturation at the same light intensity occurs earlier. In *Rba.* *sphaeroides*, where no attached cytochrome subunit with four heme groups exists, this saturation can be observed within 1 ms (Fig. 2B). The photooxidation of all strains is limited to 1 cyt *c*^3+^/RC if the interquinone electron transfer is blocked by the inhibitor terbutryne. The *pufC* strain of *Rvx.* *gelatinosus*, in which the gene encoding the RC-attached cytochrome subunit is deleted, demonstrates low level of photo-oxidation probably due to cytochromes binding alternatively to the RC (Fig. 2A). The analysis of cytochrome oxidation kinetics upon continuous strong light excitation shed light on the size of the cytochrome pool and of the availability of the cytochromes to reduce P^+^.

### (3) Flash-induced cytochrome oxidation steps

Although the investigation of the complex kinetics of cytochrome oxidation under continuous illumination has technically modest difficulty, the evaluation is limited by several complications. The duration of light excitation may coincide with or exceed the turnover times of both the reaction center and the periplasmic cytochromes. This means multiple cycles are possible within the illumination, and therefore, that the evaluation of these results should accommodate the kinetics of exchange of quinones and electrons with the RC and the cytochrome *bc*_1_ complex, respectively. The observed kinetics are a mixture of electron transfer properties from both the acceptor and donor sides. For example, if the electron flow on the acceptor side is not fast enough, this could limit the observed electron flow attributed to the donor side. Thus, the separation of the cytochrome oxidation from acceptor side effects is not straightforward. These obstacles can be largely avoided by choosing an appropriate sequence of saturating flashes for excitation.

**Figure 3:**
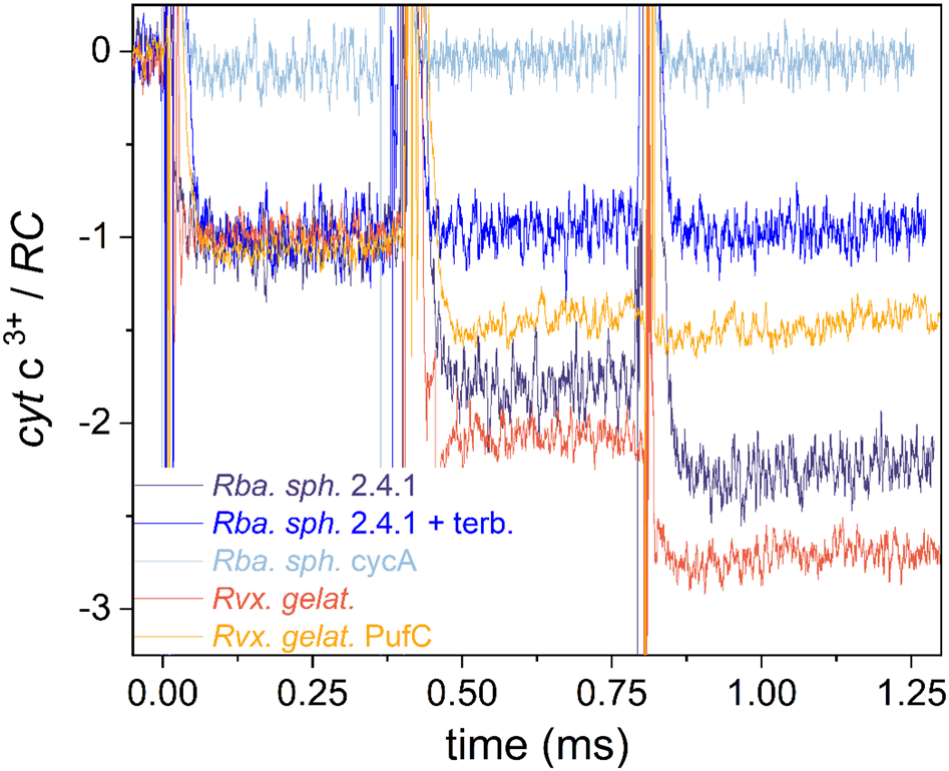
Cytochrome oxidation steps during three closely-spaced (400 μs) saturating flashes in intact cells of various wild type and mutant photosynthetic bacteria are shown. The cytochrome oxidation is measured by monitoring the absorption change at 551 nm relative to 540 nm. An average of 32 traces with 0.2 Hz repetition rate is shown. The generalized cytochrome binding constants before the second and third flashes are derived from the damping of the steps (see the model in the “Discussion”). These are *K*_D2_ = 49 and *K*_D3_ = 3.7 (*Rvx.* *gelatinosus* wild type), *K*_D2_ = 3.5 and *K*_D3_ = 1.4 (*Rba.* *sphaeroides* wild type) and *K*_D2_ = 0.75 and *K*_D3_ = 0.3 (*Rvx.* *gelatinosus* *PufC* mutant).

The proportion of oxidized cytochromes during three subsequent saturating flashes increases in a step-like function in the millisecond timescale (Fig. 3). The level of oxidized cytochromes in intact cells of *Rvx.* *gelatinosus* after 3 flashes approaches the limit of 3 cyt c^3+^/RC indicating that there is a large pool of reduced cytochromes available. In *Rba.* *sphaeroides* 2.4.1, the pool is somewhat smaller, and fewer oxidized cytochromes are so generated.

The absorption changes are abrupt after all flashes (the rise cannot be resolved in this time scale) indicating a very fast oxidation of the cytochromes independent of the flash number. The step form includes a stable state i.e., no significant further rise or decline immediately after the flash can be observed. There is no sign of slower oxidation/re-reduction that might originate from cytochromes of distal positions under diffusion control. This pattern indicates that only cytochromes in proximal positions (*i.e*., attached to the RC) participate in the observed oxidation. The oxidized cytochromes are re-reduced by the cytochrome *bc*_1_ complex later in a much longer (~ 10 ms) time scale or longer still (~ 100 ms) if the cytochrome *bc*_1_ complex is blocked by myxothiazol (see Fig. S4).

The stepwise oxidation of donor cytochromes exhibits a damping effect characteristic of different bacterial strains. *Rvx.* *gelatinosus* demonstrates the smallest damping due to the large pool of cytochromes that are available to P^+^. Significantly larger damping is observed for *Rba.* *sphaeroides* 2.4.1 that lacks a bound cytochrome subunit but that has an active soluble cytochrome *c*. The *pufC* mutant of *Rvx.* *gelatinosus* presents significantly decreased steps after the first flash as might be predicted in the absence of the RC-bound cytochrome subunit but in the presence of periplasmic cytochromes (cyc*A* and/or cyc*Y*). The addition of the quinone inhibitor terbutryne preserves the first step but eliminates the further steps in accordance with the transfer of a single electron but blockage of subsequent electron transfers on the acceptor side. The cyc*A* cytochrome deletion mutant of *Rba.* *sphaeroides* is used as an internal calibration point to normalize the level of 1 cyt c^3+^/RC since cytochrome electron transfer has been eliminated in this strain.

### (3a) Acceptor side (quinone-) dependent reactions

**Figure 4:**
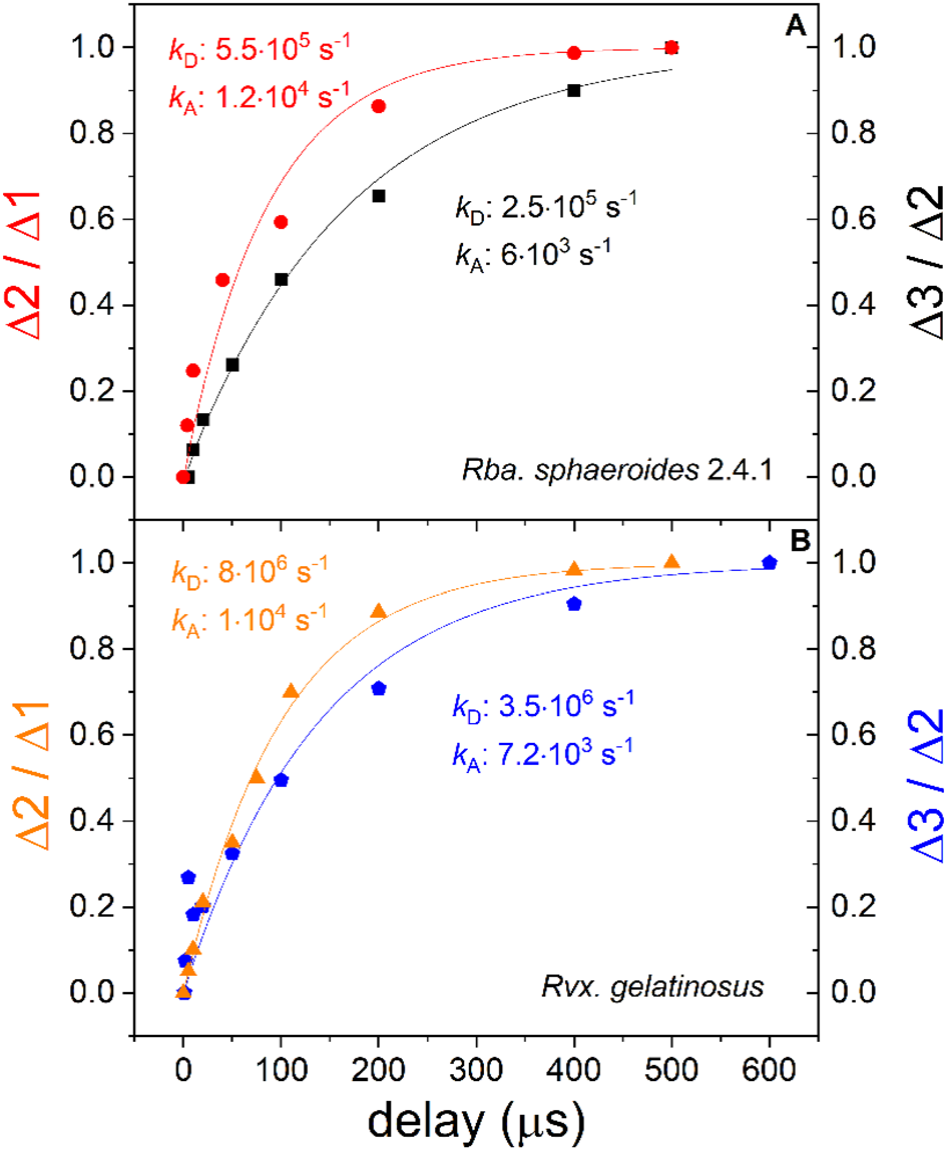
Donor (*k*_D_) and acceptor (*k*_A_) side reactions determining the reopening of the closed RC after the first and second saturating flashes in intact cells of two strains of purple photosynthetic bacteria *Rba.* *sphaeroides* (**A**) and *Rvx.* *gelatinosus* (**B**). The ratio (damping) of the magnitudes of the subsequent cytochrome oxidation steps was plotted as a function of the delay between the relevant flashes. The best-fit curves to the data were obtained from Eq. (1).

The subsequent flashes cannot be fired too close to each other because the primary quinone (Q_A_^─^) will not have completed the interquinone electron transfer to Q_B_, and thus oxidation of the special pair cannot occur, and trivial damping will be observed. By measurement of the damping of the cytochrome oxidation, the characteristic electron transfer rates can be determined. The second and third flashes relative to the first and second flashes, respectively, were fired with variable delay and the observed drop of the steps (Δ2/Δ1 and Δ3/Δ2) were plotted against the time delay (Fig.  4). Although the re-opening of the closed RC (P^+^Q_A_^─^ → PQ_A_) depends on the rates of both the donor (*k*_D_) and acceptor (*k*_A_) side reactions, *k*_A_ will be the bottle neck. In intact cells of *Rba.* *sphaeroides* 1.2·10^4^ s^─1^ rate constant (86 μs half time) was measured for the first interquinone electron transfer and 6·10^3^ s^─1^ rate constant (170 μs half time) was measured for the second interquinone electron transfer. Similar values were obtained in whole cells of *Rvx.* *gelatinosus*: 1·10^4^ s^─1^ rate constant (100 μs half time) for the first interquinone electron transfer and 7.2·10^3^ s^─1^ rate constant (140 μs half time) for the second interquinone electron transfer. The fact that these two strains exhibit such analogous values indicates the similar structure and function of the acceptor quinone sides despite significant difference on the donor side.

### (3b) Donor-side dependent reactions

**Figure 5:**
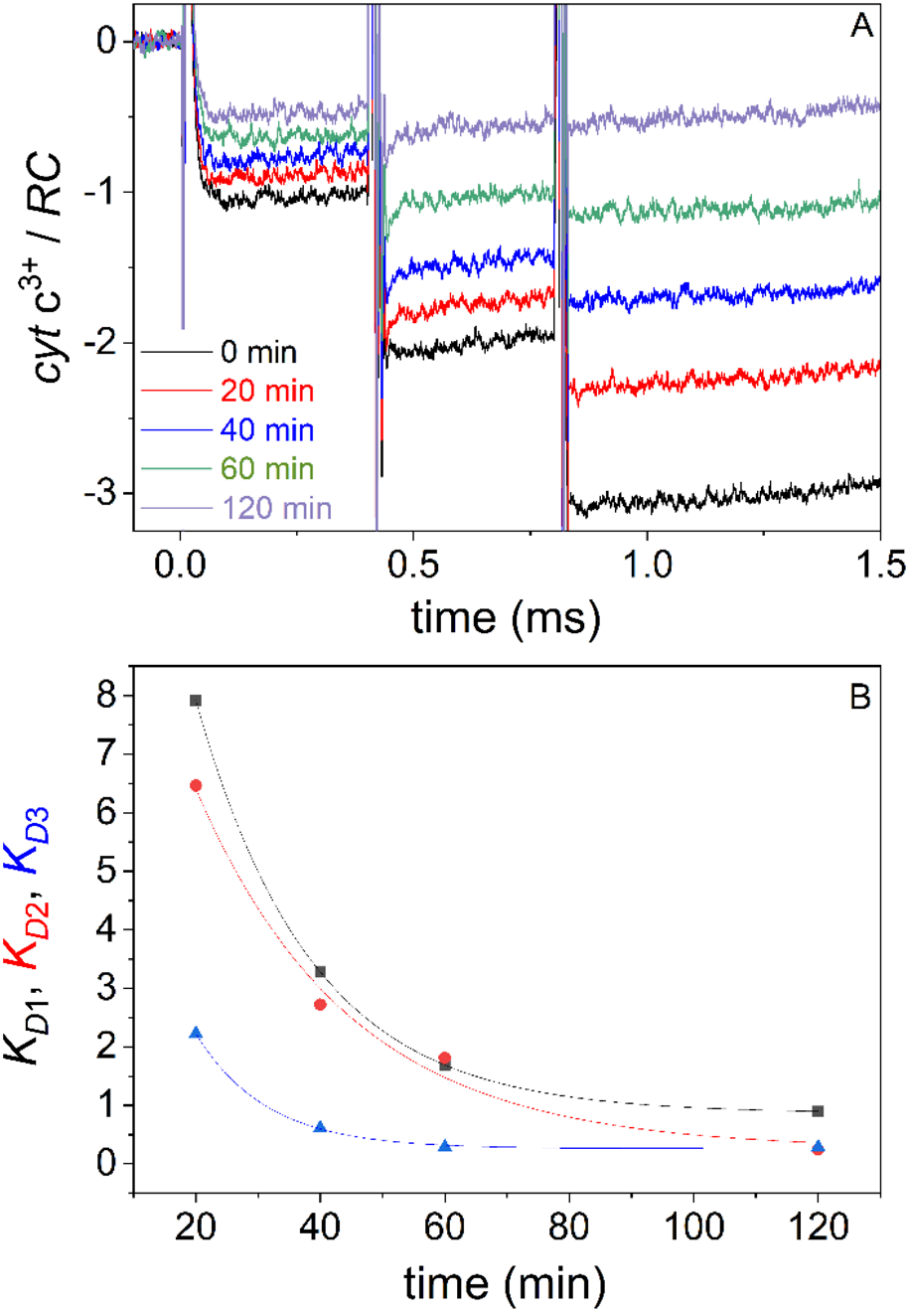
Detection of cytochrome oxidation steps while the intact cells of *Rvx.* *gelatinosus* are slowly oxidized by potassium ferricyanide (**A**). The availability of reduced cytochromes expressed by equilibrium constants *K*_D1_, *K*_D2_ and *K*_D3_ after the first, second and third flash, respectively were calculated from Eqs. (5)–(7) and plotted against the time of incubation with potassium ferricyanide (**B**). The bacteria were grown 48 h in the culture and the oxidation of the cells was initiated by addition of potassium ferricyanide of 1 mM concentration and monitored for 2 h.

By use of external redox mediators, the pattern of cytochrome oxidation steps can be selectively modified. The addition of the reducing agent dithionite will eliminate cytochrome oxidation as a consequence of reduction of the quinone acceptor site. The oxidizing agent potassium ferricyanide, however, has a much more sophisticated effect due to its slow uptake by the cell and to the very slow and progressive establishment of a redox equilibrium between the low potential hemes in PufC in *Rvx.* *gelatinosus*. A similar effect was observed in *Bl.* *viridis*^30^. Although the actual redox potential inside the cell cannot be monitored, the persistent establishment of a redox equilibrium in the presence of ferricyanide makes it possible to probe the damping we observed in the cytochrome oxidation steps (Fig. 5). The gradual oxidation by potassium ferricyanide will modify the donor side only but the acceptor side will remain unaffected given the unavailability of the acceptor side to an ionic agent like potassium ferricyanide. Thus, by increasing time after ferricyanide addition, the cytochromes will be gradually oxidized, and thus fewer reduced cytochromes will be available for light-induced oxidation. This process is expressed by a gradual increase of the damping of the steps including the first steps, relative to untreated cells as well. The observation offers clear-cut support that not the acceptor side but the availability of reduced cytochromes to the RC determines the damping of the cytochrome oxidation steps under conditions used in these experiments.

### (4) P^+^ re-reduction kinetics and effect of external donor ferrocene

**Figure 6:**
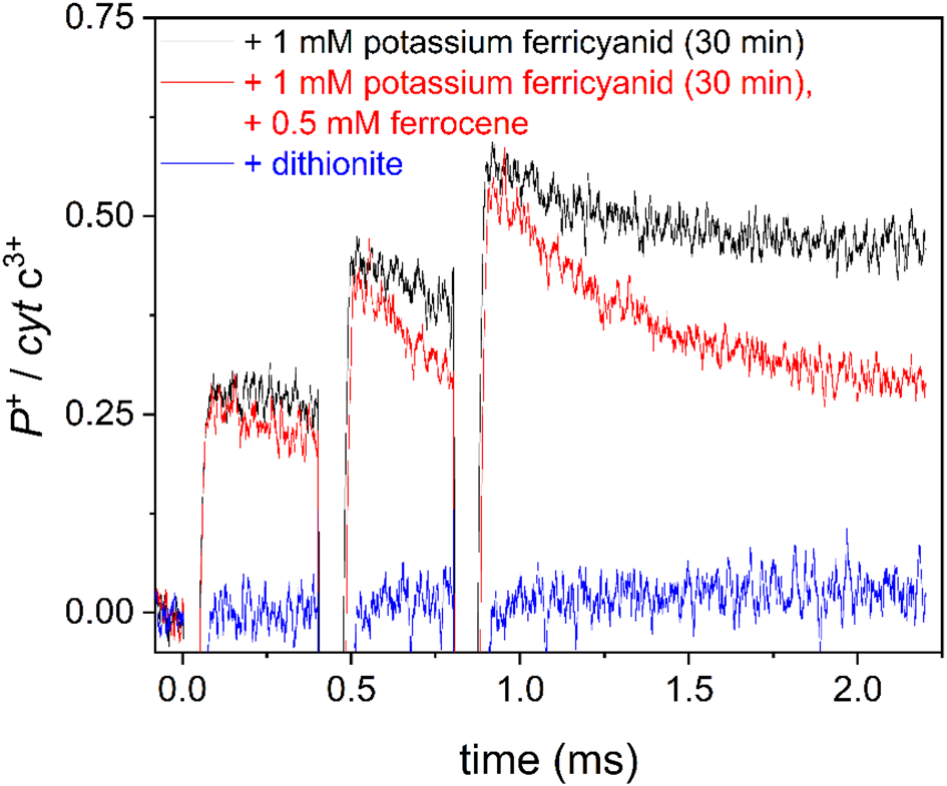
Trains of flash-induced oxidized dimer (P^+^) in intact cells of *Rvx.* *gelatinosus* treated by an oxidizing agent potassium ferricyanide (1 mM) for 30 min in the absence (black) and presence (red) of an externally added electron donor ferrocene in large excess (0.5 mM). As a reference, the addition of the reducing agent dithionite (blue) prevented the accumulation of the oxidized dimer since the acceptor quinone side became reduced. Similarly, partial oxidation of the cytochromes enabled a detectable amount of P^+^. P^+^ was quantified spectroscopically by following absorption changes upon oxidation of TMPD by P^+^ in cytochrome mutants (see “Materials and methods”) and is shown relative to the concentration of oxidized cytochromes.

The cytochromes are oxidized (cyt *c*_2_^2+^  → cyt *c*_2_^3+^) directly by the light-oxidized RC dimer P^+^ which in turn will be re-reduced (P^+^  → P). Thus, we thought it worthwhile follow the coupling of these two redox couples by monitoring the P^+^ signal via absorption change at 790 nm relative to 750 nm, and concomitant with cytochrome oxidation. The calibration of the P^+^ signal with TMPD in cytochrome deficient *cycA* cells (see “Materials and methods”) enables the quantitative comparison of P^+^ with cyt *c*_2_^3+^. In wild-type strains, only a negligible amount of P^+^ remains due to the very effective electron donation by the cytochromes. However, in mutants or in wild type cells with cytochromes partly reduced by chemical means, a significant amount of oxidized dimer can be observed (Fig. 6). An increasing amount of P^+^ is detectable after multiple flashes mirroring the damping of cytochrome oxidation we observed. The P^+^ levels are not as stable as those observed for the cytochrome oxidation steps, revealing the effect of a relatively slow external electron donation of unknown origin in the cell. The re-reduction of P^+^ can be accelerated in the sub millisecond time scale by the addition of a large excess (0.5 M) of the external electron donor ferrocene. As the ferrocene is an uncharged redox agent, it can easily penetrate the cell wall and get access to the interior of the bacterium. Because reduction of P^+^ by ferrocene is collisional in nature, and the concentration of P^+^ is larger after the third flash than after the first flash, the rate constant for diffusion-controlled re-reduction by ferrocene must also be larger after the first flash than the second flash. Thus, the accumulation of P^+^ favours its reduction, and a slightly faster decay (increased slope) of the P^+^ signal can be observed after each subsequent flash (see Fig. 6).

## Discussion

The novelty of the present study is the detection of cytochrome oxidation in intact bacterial cells under continuous and flash illumination. The cytochrome assay has been routinely and consistently applied in artificial systems of isolated RCs with externally added cytochromes. But to our knowledge, it has never been used for whole cells with their natural cytochromes probably due to experimental difficulties. The conditions of the cytochrome turnover in isolated RCs can be easily adjusted to assure the exclusive domination of the acceptor side loss processes causing the damping of the cytochrome steps^31^. We will demonstrate here that the cytochrome assay can be applied for intact systems, as well, and the pattern of the observed cytochrome oxidation is controlled by the loss processes on both sides of the RC. The discussion will focus on the understanding of the damping of the cytochrome oxidation and what information can be obtained for loss parameters on the donor side.

**Figure 7:**
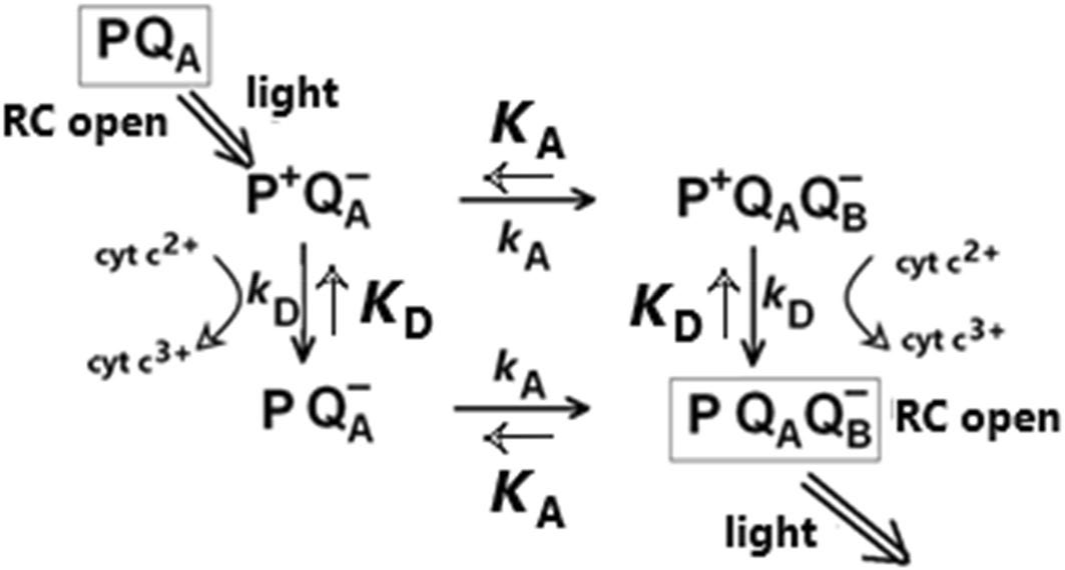
A reaction scheme for the first flash-induced closing and opening of the RC is shown with light excitation and dark electron transfer processes indicated. The RC is closed by a flash and can be re-opened by simultaneous donor and acceptor side reactions having rate constants k_D_ and *k*_A_, and equilibrium constants *K*_D_ and K_A_. This scheme can be considered as a link in a chain of reaction schemes. Since both reactions depend on the redox state of the acceptor complex that can hold between 0 and 3 electrons, the second and third flash would show the semi-reduced (Q_A_^─^ and Q_B_^─^ and fully-reduced Q_B_H_2_) states, respectively. The various RC states under continuous illumination or after subsequent saturating flashes can be described by coupling these links.

The observed kinetics and stoichiometry of the oxidation of cytochromes are closely related to the turnover of the RC. Illumination in the form of either continuous or flash excitation closes the RC which will be re-opened after a series of dark reactions both on the donor and the acceptor sides. A single link of a chain of reactions for the first flash is summarized in Fig. 7. Because of the large values of the reaction rate constants (> (1 ms)^−1^), the influence of the cytochrome *bc*_1_ complex is negligible (see Fig. S4). The quinone acceptor can accumulate 3 electrons at most before quinone exchange at the Q_B_ site, so the entire reaction scheme would consist of a chain of three such links with one, two, or three electrons in the acceptor complex. The reactions on the donor side are more complex. In mutants that lack a cytochrome subunit (*Rba.* *sphaeroides*), the oxidation of the cytochromes by P^+^ requires two consecutive events: binding of the reduced cytochrome to the docking site of the RC followed by electron transfer between the redox partners P^+^ and cyt *c*_2_^2+^. As soon as the cytochrome becomes oxidized, it should leave the docking site and should be exchanged by a reduced one from the cytochrome pool. Gerencsér et al*.* mixed horse heart cytochrome *c* and detergent-solubilised RC-only complexes in solution and showed that dissociation of the ET complex is the bottleneck of cytochrome turnover, and the exchange of the cytochrome is a product-inhibited process^32^. Several other studies showed preferential binding of oxidized cytochrome *c*_2_ to RCs^33^, to the extent that the oxidized cytochrome impeded the access of reduced cytochrome *c*_2_ to its docking site on the RC. Using single molecule force spectroscopy, surprisingly large forces between 112 and 887 pN were required to pull apart the P–oxidized cytochrome *c*_2_ complex^34^. They found a duration of milliseconds for the persistence of the post-ET oxidized cytochrome *c*_2_–P “product” state compatible with rates of cyclic photosynthetic ET. Given the hydrophobic surface interaction between RC and its tetraheme subunit^20^ we speculate that non-native interactions between periplasmic cytochromes and the RC might be more transient. However, this result is not supported by our measurements: the cytochrome exchange time should be smaller because subsequent saturating flashes with delay of 400 μs were already able to turn over the RC completely.

If a tetraheme cytochrome subunit is firmly attached to the RC (*Rvx.* *gelatinosus*), the exchange of the periplasmic reduced cytochrome *c*_2_ will occur at the docking site of the subunit. In all cases, the bottle neck of the accumulation of oxidized cytochromes is the availability of the reduced cytochromes including both exchange and electron transfer processes.

In the case of continuous and strong illumination, the solution of the linear differential (rate) equations derived from consecutive coupling of a chain of reaction schemes with 0 to 3 electrons in the acceptor complex offers kinetics with characteristic phases (photochemical, electron-transfer limited and saturation). This is similar to what we have demonstrated experimentally (Fig. 2). Because the light excitation is continuous, the system must be eventually driven from the dark-adapted state to its maximum capacity, i.e., 3 electrons in the acceptor side and 3 cyt c_2_^3+^/RC on the donor side (see Fig. S3).

In the case of the flash excitation experiments, we have applied the model based on the initial branching and final unification of the symmetrical reaction paths as described in Fig. 7. The kinetics of the re-opening of the RC therefore includes the two rate constants *k*_D_ and *k*_A_ symmetrically. The analytical form can be obtained from the solution of the set of linear differential equations with proper initial conditions:

1 $$\left[{\mathrm{PQ}}_{\mathrm{A}}\right]\left(t\right)=\left(1-{e}^{-{k}_{A}t}\right)\left(1-{e}^{-{k}_{D}t}\right)$$

Here *k*_A_ and *k*_D_ denote the rate constants assuring the opening the RC having different redox states on the acceptor and donor sides, respectively. As expected, Eq. (1) is symmetric to the two sides and implies that the contribution of both sides is required to re-open the RC. The decomposition of the kinetics of cytochrome oxidation (*i.e.* the increase of cyt *c*_2_^3+^ ) by delay of the second flash relative to the first (or the third relative to the second) according to Eq. (1) enables the separation of the contributions of the acceptor and donor-sides (Fig. 4). Our results show that the acceptor side reactions were the rate limiting steps both in the one and in the two electron states in the bacterial strains studied here. The first interquinone electron transfer is a factor of 2 faster (86 μs) than the second interquinone electron transfer (170 μs). This method of delayed flashes delivers reliable values for the first and second interquinone electron transfer times in intact cells of photosynthetic bacteria hardly obtained by any other means.

The stationary solution of the set of linear differential equations of the scheme in Fig. 7 describes the steps of cytochrome oxidation measured in our experiments. Using the thermodynamic box of the energy levels of the different RC states originating from branching and unification of the electron pathways, we obtain for each period between flashes:

2 $$\Delta 1=\frac{{K}_{D1}}{1+{K}_{D1}}$$

3 $$\Delta 2=\left[\frac{{K}_{A1}}{1+{K}_{A1}}\right]\left[\frac{{K}_{D1}}{1+{K}_{D1}}\cdot \frac{{K}_{D2}}{1+{K}_{D2}}\right]$$

4 $$\Delta 3=\left[\frac{{K}_{A1}}{1+{K}_{A1}}\cdot \frac{{K}_{A2}}{1+{K}_{A2}}\right]\left[\frac{{K}_{D1}}{1+{K}_{D1}}\cdot \frac{{K}_{D2}}{1+{K}_{D2}}\cdot \frac{{K}_{D3}}{1+{K}_{D3}}\right]$$

where Δ*i* denotes the step of cytochrome oxidation after the *i*th flash (*i* = 1, 2 or 3), *K*_Di_ and *K*_Ai_ are the donor and acceptor side equilibrium constants, respectively. *K*_Di_ includes the binding/unbinding (exchange) and the subsequent oxidation of cytochromes after the *i*th flash i.e., in the state of *i* electrons on the acceptor sides (or *i*-holes on the donor side). For example, *K*_D1_ = [PQ_A_^─^Q_B_]/[P^+^Q_A_^─^Q_B_] = [PQ_A_Q_B_]/[P^+^Q_A_Q_B_^─^]. *K*_Ai_ expresses the electron equilibrium constants between Q_A_ and Q_B_ if the acceptor side is reduced by one (*i* = 1) or by two (*i* = 2) electrons (*K*_A_ refers to the re-oxidation of Q_A_^─^).

Equations (2)–(4) indicate clearly that the steps depend on the equilibrium constants on both the acceptor and the donor sides. Their contributions can be separated and further simplified: the equilibrium constants on the acceptor side are much larger than 1 at each flash: *K*_A1_ >  > 1 and *K*_A2_ >  > 1. This comes from the facts that the turnover of the acceptor side is highly effective due to bound quinones (no docking is required before the electron transfer) and to the large redox gap (large driving force) for the electron transfer. Consequently, the terms referred to the acceptor side (the first bracket) can be neglected (taken as 1) and the damping of the oxidation steps can be attributed exclusively to *K*_D_’s, to the availability of cytochromes (second bracket) which can be expressed by the measured cytochrome oxidation steps Δ1, Δ2 and Δ3:

5 $${K}_{D1}=\frac{\Delta 1}{1-\Delta 1}$$

6 $${K}_{D2}=\frac{\frac{\Delta 2}{\Delta 1}}{1-\frac{\Delta 2}{\Delta 1}}$$

7 $${K}_{D3}=\frac{\frac{\Delta 3}{\Delta 2}}{1-\frac{\Delta 3}{\Delta 2}}$$

*K*_D_ serves as a quantitative measure of the availability of reduced cytochromes. With increasing flash number, the level and therefore the availability of the reduced cytochromes progressively decreases which is reflected by the gradual decrease of the *K*_D_ values. Indeed, this tendency is observed in our experiments. Due to the correlation between *K*_D_ and the availability of reduced cytochrome, the drop of *K*_D_ was observed upon increase of the flash number (Fig. 3) or by titration with the oxidizing redox agent potassium ferricyanide (Fig. 5).

In this work, we offer a significant extension of the standard “cytochrome assay” used to detect the photochemical activity of the RC in artificial systems (*i.e.,* isolated RCs and cytochromes in vitro). Our methods and results enable the quantitative description of the availability of reduced cytochromes to the RC, which in turn determines the capacity of the cytochrome pool to re-reduce the oxidized P^+^ dimer. By analysis of the damping, we observed in successive steps of flash-induced cytochrome oxidation, we could get closer to a solution of the long-debated structural and functional questions surrounding electron transfer between the RC and periplasmic donor cytochromes in different strains and mutants of intact, whole cells of photosynthetic bacteria.

## Materials and methods

### Bacterial strains, growth conditions and culture additions

Bacterial strains and plasmids and oligonucleotide primer sequences used in this work are listed in Tables 1 and 2, respectively.

**Table 1:** Bacterial strains and plasmids.

	Relevant characteristics	Source/References
**A. Strains**		
*E.* *coli*		
DH5α	*supE44* *lacU169(480lacZDM15)* *hsdR17* *recA1* *endA1* *gyrA96* *thi-1* *relA1*	^43^
JM109	*endA1,* *recA1,* *gyrA96,* *thi,* *hsdR17* *(rk*^*−*^*,* *mk*^+^*),* *relA1,* *supE44,* *Δ(lac-proAB),* *[F′* *traD36,* *proAB,* *laqI*^*q*^*ZΔM15]*	^39^
S17-1	*TpR* *SmR* *recA,* *thi,* *pro,* *hsdR-M* + *RP4:* *2-Tc:Mu:* *Km* *Tn7* *λpir*	^44^
*Rba.* *sphaeroides*		
2.4.1	Wild-type	^35^
JS2293Δ	In-frame *cycA* deletion	This study
JS2302Δ	In-frame *cycA* deletion; in-frame *cytC4* deletion	This study
*Rvx.* *gelatinosus*		
Wild-type	Wild-type	^11^
JS2315Δ	In-frame *pufC* deletion	This study
**B. Plasmids**		
pUC19	Cloning vector	^45^
pKanMobSacB	Suicide vector	^46^
pJS280	pUC19::*cycA*	This study
pJS281	pUC19::*cytC4*	This study
pJS287	pUC19::*cytC4* with upstream *Bam*HI site-directed mutation	This study
pJS294	pJS287 with downstream *Bam*HI site-directed mutation	This study
pJS286	pJS280 *Bam*HI site-directed mutation	This study
pJS292Δ	pJS286 *Bam*HI in-frame deletion	This study
pJS293Δ	pKanMobSacB::*cycA* in-frame deletion	This study
pJS297Δ	pJS287 *Bam*HI in-frame deletion	This study
pJS302Δ	pKanMobSacB::*cytC4* in-frame deletion	This study
pJS314Δ	pBluescript::*pufC* in-frame deletion	This study
pJS315Δ	pKanMobSacB::*pufC* in-frame deletion

**Table 2:** Oligonucleotide primer sequences.

Name	Sequence	Notes/use
cycA_F	5’ AATTGAATTCGCAGTAGTGATTGTGTGCCG 3’	Cloning of wild-type *cycA* gene
cycA_R	5’ GGCTGCAGCTGAATGTACTCACCGCCCC 3’	Cloning of wild-type *cycA* gene
cycA_SDM_F	5’ GCGACCCGGAtcCCGGGGCCAAG 3’	SDM of *cycA* to introduce *Bam*HI site
cycA_SDM_R	5’ CTTCCTGCGCGAGCGCCG 3’	SDM of *cycA* to introduce *Bam*HI site
cycA_SEQ_F	5’ GTATCCGGGCAGCATAGTCC 3’	Sequence analysis of *cycA* deletion
cycA_SEQ_R	5’ CGTGATCTCCTATCTGCGGG 3’	Sequence analysis of *cycA* deletion
cytC4_F	5’ AATTGAATTCCCGCACCAACGAATACAAGG 3’	Cloning of wild-type *cytC4* gene
cytC4_R	5’ GGCTGCAGCTTCACGCTCTCCTTCGTGG	Cloning of wild-type *cytC4* gene
cytC4_SDM_UP_F	5’ TCTGGGCGGGatcCTCCTCGCCC 3’	SDM of *cytC4* to introduce *Bam*HI site
cytC4_SDM_UP_R	5’ CCGGCCAGAAAGCCAAGCC 3’	SDM of *cytC4* to introduce *Bam*HI site
cytC4_SDM_DN_F	5’ GGCAAGCGGAtcCACGAGATCATGTCGC 3’	SDM of *cytC4* to introduce *Bam*HI site
cytC4_SDM_DN_R	5’ GGTGCGGAAGGCCTTGAG 3’	SDM of *cytC4* to introduce *Bam*HI site
cytC4_SEQ_F	5’ TCTGGTTCACCGACAATCAGG 3’	Sequence analysis of *cytC4* deletion
cytC4_SEQ_R	5’ TTTATGCAGGGTCTGTCCCC 3’	Sequence analysis of *cytC4* deletion
pufC_LHS_F	5’CTCGAGTGGCTGTATCTGGTGCTGG 3’	Cloning of *pufC* upstream region
pufC_LHS_R	5’ GAATTCGGTCGAAATGCGAACCGC 3’	Cloning of *pufC* upstream region
pufC_RHS_F	5’ GGATCCCGAAGCCCGCGTCAGC 3’	Cloning of *pufC* downstream region
pufC_RHS_R	5’ GAATTCACCGCCGCGAAGTAACG 3’	Cloning of *pufC* downstream region
pufC_SEQ_F	5’ GGCGGAAAAAGAAGAAGCCG 3’	Sequence analysis of *pufC* deletion
pufC_SEQ_R	5’ CAACTGGTATCTGTGGGCCG 3’	Sequence analysis of *pufC* deletion

Underlined, restriction site incorporated into the primer.

Lower-case, deviation from wild-type sequence.

Purple nonsulfur photosynthetic bacteria *Rba.* *sphaeroides* strain 2.4.1^35^ and *Rvx.* *gelatinosus* wild type^12^ were grown for spectrophotometric analysis in Siström’s medium^36^ in completely filled screw top vessels without oxygen (anaerobic growth) under irradiance of 13 W m^−2^ from tungsten lamps^27^. The cells were harvested at an exponential phase of the growth at cell concentration of ~ 10^8^ cell mL^–1^ and were bubbled with nitrogen for 15 min before measurements to preserve the anoxic conditions.

Otherwise, *Rba.* *sphaeroides* strains were grown aerobically at 30 °C in YCC medium^37^ and *Rvx.* *gelatinosus* was grown aerobically at 30 °C in RCV medium^38^. Media was supplemented when appropriate with kanamycin (50 μg mL^–1^), fructose (30 mg·mL^─1^), and DMSO (120 mM). Conjugal transfer of strains from *E.* *coli* to *Rba.* *sphaeroides* was performed as described previously, and counter-selection against S17-1 conjugal donors was achieved by addition of tellurite (100 μg mL^–1^)^39^. *Escherichia* *coli* strains were grown at 37 °C in LB medium^31^ supplemented with antibiotics when appropriate; kanamycin (50 μg mL^–1^) and ampicillin (100 μg mL^–1^).

### Construction of in-frame deletions of cycA, cytC4, and pufC

The deletions of portions of genes encoding periplasmic cytochromes were planned using published cytochrome crystal structures^19,40^ and molecular modeling by Phyre2^41^, to identify functionally critical residues. Strain JS2293Δ, containing an in-frame deletion of the *cycA* gene encoding cytochrome *c*_2_ in *Rba.* *sphaeroides* (NCBI Reference sequence WP_002720461.1), was genetically constructed essentially as described previously^42^. An 840 bp fragment of DNA containing the *cycA* reading-frame and flanking DNA was generated by PCR (Phusion polymerase, Thermo Scientific) in 5X GC Phusion Buffer using specific primers *cycA*_F and cyc_R. Digestion of the resulting PCR product with *Eco*RI and *Pst*I and ligation into similarly digested pUC19 produced plasmid pJS280. The wild-type *cycA* gene contains a single *Bam*HI site, so we introduced a second *Bam*HI site by site-directed mutagenesis such that excision of the resulting *Bam*HI fragment and re-ligation would result in an in-frame deletion of codons 29 through 105 of the *cycA* gene. This deletion was generated by PCR with mutagenic primers *cycA*_SDM_F and *cycA*_SDM_R, to produce plasmid pJS286. Following verification by dideoxy sequencing, the *Bam*HI fragment was excised and religated, resulting in plasmid pJS292Δ. The *Eco*RI-*Pst*I fragment bearing the in-frame deletion of *cycA* was transferred to the suicide vector pKanMobSacB to produce plasmid pJS293Δ. Transfer of this fragment to the chromosome of wild-type *Rba.* *sphaeroides* was accomplished as described previously^42^, except that counterselection against the JM109 donor was achieved by plating exconguates on YCC containing kanamycin and tellurite as described above. Secondary recombinants were selected by passage on YCC containing 10% sucrose, and double recombinants that yielded the deletion were screened first for loss of the kanamycin resistance marker, and were subsequently confirmed by dideoxy sequence analysis of a PCR product generated by flanking primers *cycA*_SEQ_F and *cycA*_SEQ_R.

Deletion of the *cytC4* gene (NCBI Reference sequence WP_009564385.1) was accomplished similarly: a 1.2 kb fragment of DNA containing the *cytC4* reading-frame and flanking DNA was generated by PCR using specific primers cytC4_F and cytC4_R. This PCR product was digested with *Eco*RI and *Pst*I and ligated into similarly-digested pUC19 to produce plasmid pJS281. Two in-frame *Bam*HI sites were introduced sequentially into pJS281 using mutagenic primers. First, cytC4_SDM_UP_F and cytC4_SDM_UP_R were used to introduce a *Bam*HI site on the 5’ end of the gene, to produce plasmid pJS287. This plasmid was used as template with mutagenic primers cytC4_SDM_DN_F and cytC4_SDM_DN_R, to produce plasmid pJS294. Following dideoxy sequence verification, the internal *Bam*HI segment was excised and the plasmid religated to produce plasmid pJS297Δ. As above, the *Eco*RI-*Pst*I fragment bearing the in-frame deletion of *cytC4* was transferred to the suicide vector pKanMobSacB to produce plasmid pJS302Δ. Stable recombination of the *cytC4* in-frame deletion into strain JS2293Δ produced a double deletion strain JS2302Δ. The genomic sequence of this mutant was confirmed by dideoxy sequencing of a PCR product generated by flanking primers cytC4_SEQ_F and cytC4_SEQ_R.

Deletion of the *pufC* gene in *Rvx.* *gelatinosus* (NCBI Reference sequence WP_231384551.1) was achieved by PCR amplification of two segments of DNA immediately upstream (LHS) and downstream (RHS) of *pufC.* These two segments were engineered so that when fused, an in-frame deletion of *pufC* would be constructed. The upstream LHS segment was generated with PCR primers pufC_LHS_F and pufC_LHS_R, and the downstream segment was generated with PCR primers pufC_RHS_F and pufC_RHS_R. The LHS PCR product was digested with *XhoI* and *Bam*HI, the RHS product was digested with *Eco*RI and *Bam*HI, and both pieces were cloned into pBluescript that was digested with *Xho*I and *Eco*RI, producing plasmid pJS314Δ. Following dideoxy sequence confirmation, the LHS::RHS fusion was excised from pBluescript by *Xho*I *Eco*RI digest and cloned into *Sal*I-*Eco*RI-digested pKanMobSac to produce pJS315Δ. Following mobilization of this construct to the chromosome of *Rvx.* *gelatinosus* as described above, correct construction of strain JS2315Δ was verified by sequencing a PCR product generated with primers pufC_SEQ_F and pufC_SEQ_R.

*Rvx.* *gelatinosus* grows well under respiratory conditions (about 2 h of the doubling time) as well as under photosynthetic conditions (about 2.5 h of the doubling time). The photosynthetic growth rate of the *Rvx.* *gelatinosus* mutant lacking the cytochrome subunit (PufC) was roughly estimated to be about a half of that of the wild type, showing that the cytochrome subunit is not essential but is advantageous for photosynthesis.

### Chemicals

Terbutryne was used at 120 μM concentration to block the Q_A_ → Q_B_ interquinone electron transfer in the RC as terbutryne competes with quinone for the Q_B_-binding site. Myxothiazol was used in 10 μM concentration to inhibit the cyclic electron transfer, as it is a competitive inhibitor of ubiquinol and binds at the quinol oxidation (Q_o_) site of the cytochrome *bc*_1_ complex.

### Preparation of chromatophores

Photosynthetic membranes of chromatophores of wild-type and mutant purple non-sulphur bacteria *Rb.* *sphaeroides* were isolated from fresh cells of a 5–6-day-old culture washed with sodium phosphate buffer (100 mM, pH 7.5). The cells were broken by ultrasonic disintegration followed be fractional centrifugation. The intact cells and large particles were separated by centrifugation at 40,000 g for 15 min. The chromatophores obtained by centrifugation of the supernatant at 144,000 g for 120 min were suspended in 50 mM sodium phosphate buffer (pH 7.5). Before measurements, they were diluted with the buffer to a concentration corresponding to ~ 10 μM photoactive pigment.

### Light‑induced absorption changes

The experiments were carried out by a home-constructed spectrophotometer^35^ with some modifications. The absorption changes were evoked either by high power (2 W) laser diodes (typical wavelength of 808 nm, Roithner LaserTechnik LD808-2-TO3) of variable duration (up to 20 ms) or by a train of three saturating Xe flashes of duration 3 μs fired close (400 μs) to each other. Each Xe flashes (alone) were saturating checked by choosing no delay among the flashes. After passing through appropriate cut-off glass filters, the Xe flashes illuminated the sample in a 1 × 1 cm quartz cuvette from three different directions (see Fig. S1). A stabilized 130 W tungsten lamp was the light source of the measuring light whose wavelength and bandwidth were selected by a monochromator (Jobin–Yvon H-20 with concave holographic grating). The transmitted measuring light was detected by a photomultiplier (R928 Hamamatsu) protected from the scattered exciting light by filters. The detector was connected to a differential amplifier and to a digital oscilloscope (Tektronix TDS 3032). The time resolution of the device was limited to 50 μs. The light-induced signals of the chromophores or intact cells measured at characteristic wavelengths were always related to those at reference wavelengths.

### P/P^+^ related absorption change coefficient

To quantify the amount of light-induced oxidized P^+^ dimer in wild-type strains, flash-induced absorption changes of the cytochrome-less *cycA* mutant were measured in the absence (P/P^+^: 790 nm vs. 750 nm) and in the presence of the electron donor TMPD. (TMPD/TMPD^+^: 611 nm vs. 675 nm). Using a known absorption change coefficient for TMPD (Δε_TMPD_ = 12.2 mM^−1^ cm^−1^), we obtained Δε_P/P+_  = 70 ± 5 mM^−1^ cm^–1^. The amount of oxidized cytochrome was determined from the absorption change coefficient Δε(cyt *c*^2+^/cyt *c*^3+^ (551–540 nm)) = 21.1 mM^−1^ cm^−1^^26^.

The intact cells were re-suspended in fresh medium, anaerobically adapted in the dark and bubbled with nitrogen for 15 min and enclosed by a glass cap to permit flash excitation from the top prior to measurement (see Fig. S1). To increase the signal-to-noise ratio of the absorption changes, the average of 32 traces with repetition rate of (0.2 s)^−1^ were taken. All measurements were done at room temperature.

## Supplementary Information

Supplementary Figures.

## Data Availability

Data generated by dideoxy sequence analysis of the in-frame deletions utilized in this study have been deposited to the Sequence Read Archive (SRA) at the National Center for Biotechnology Information, with Bioproject accession numbers as follows: *Rba.* *sphaeroides* *cytC4* (PRJNA847588), *Rba.* *sphaeroides* *cycA* (PRJNA847566) and *Rvx.* *gelatinosus* *pufC* (PRJNA847559).
