# Supplementary material for: Capacity and kinetics of light-induced cytochrome oxidation in intact cells of photosynthetic bacteria
Source: Sci Rep. 2022 Aug 22;12:14298. doi: 10.1038/s41598-022-18399-y (PMC9395421; doi:10.1038/s41598-022-18399-y)
Supplement: Supplementary file 1 — Supplementary Figures. [file 41598_2022_18399_MOESM1_ESM.docx]

Supplementary Information

Nature Scientific Report

revised version submitted

Capacity and kinetics of light-induced cytochrome oxidation in intact cells of photosynthetic bacteria

Mariann Kis^1,2^, James L. Smart^3^ and Péter Maróti^1*,^

^1^Department of Medical Physics and Informatics, University of Szeged, H-6720 Szeged, Hungary

^2^Balaton Limnological Research Institute, 8237 Tihany, Hungary

^3^Department of Biological Sciences, University of Tennessee at Martin, Martin, TN 38238 USA

Electronic address: kis.mariann.m@gmail.com

Electronic address: jsmart@utm.edu

Electronic address: pmaroti@sol.cc.u-szeged.hu

*Corresponding author

Address: Department of Medical Physics and Informatics, University of Szeged, Rerrich Béla tér 1. Szeged H-6720 Hungary

phone: 36-62-544-120,

e-mail: [pmaroti@sol.cc.u-szeged.hu](mailto:pmaroti@sol.cc.u-szeged.hu)


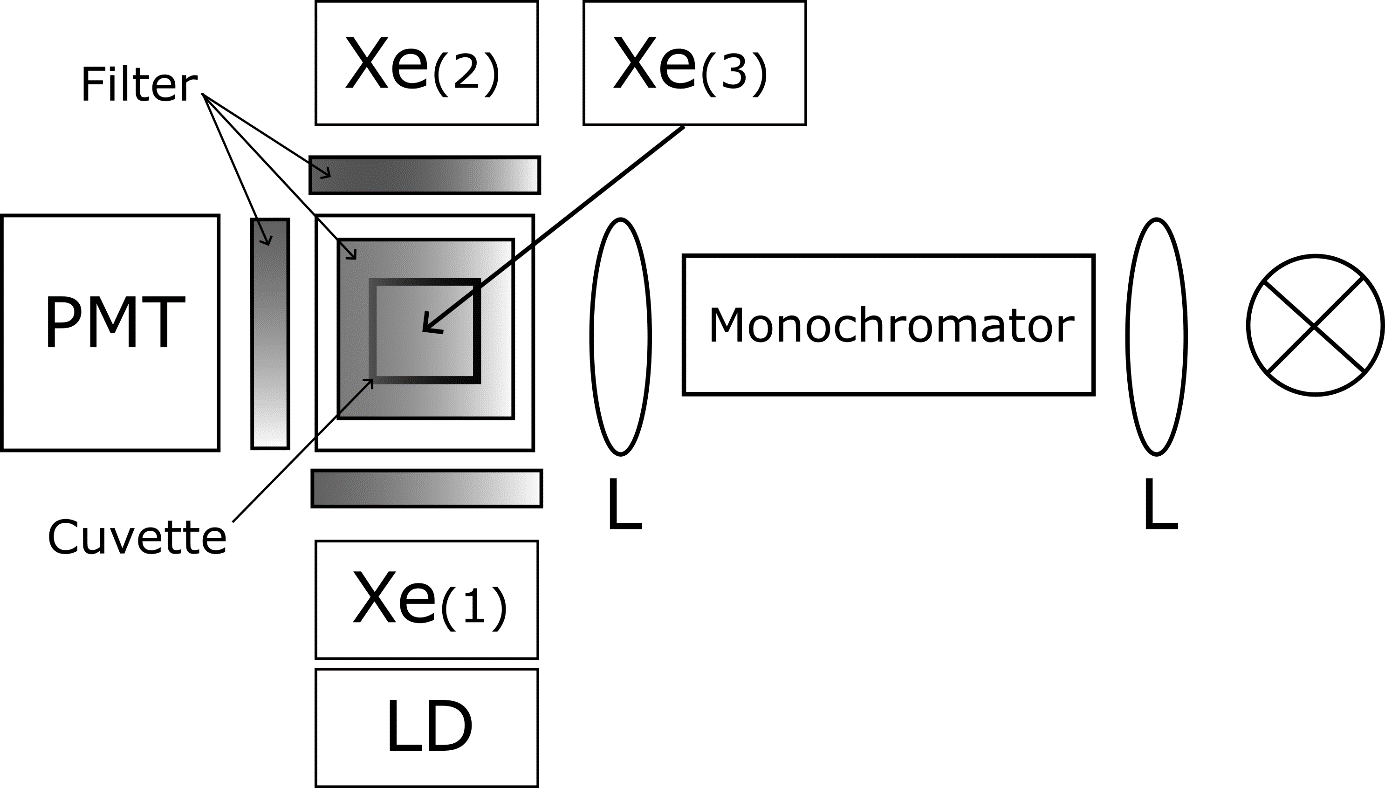


**Figure S1.** Schematic top view of the arrangement of the optical devices to measure light-induced absorption changes in whole cells of purple bacteria. The sample in the cuvette can be excited by three Xe flashes and by a laser diode (LD) perpendicular to the test beam from the DC light source. The spectral ranges of the excitation and wavelength of the monitoring beam can be selected by colour filters and monochromator (2 nm half bandwidth), respectively. The photomultiplier is protected by colour filters crossed with the excitation.


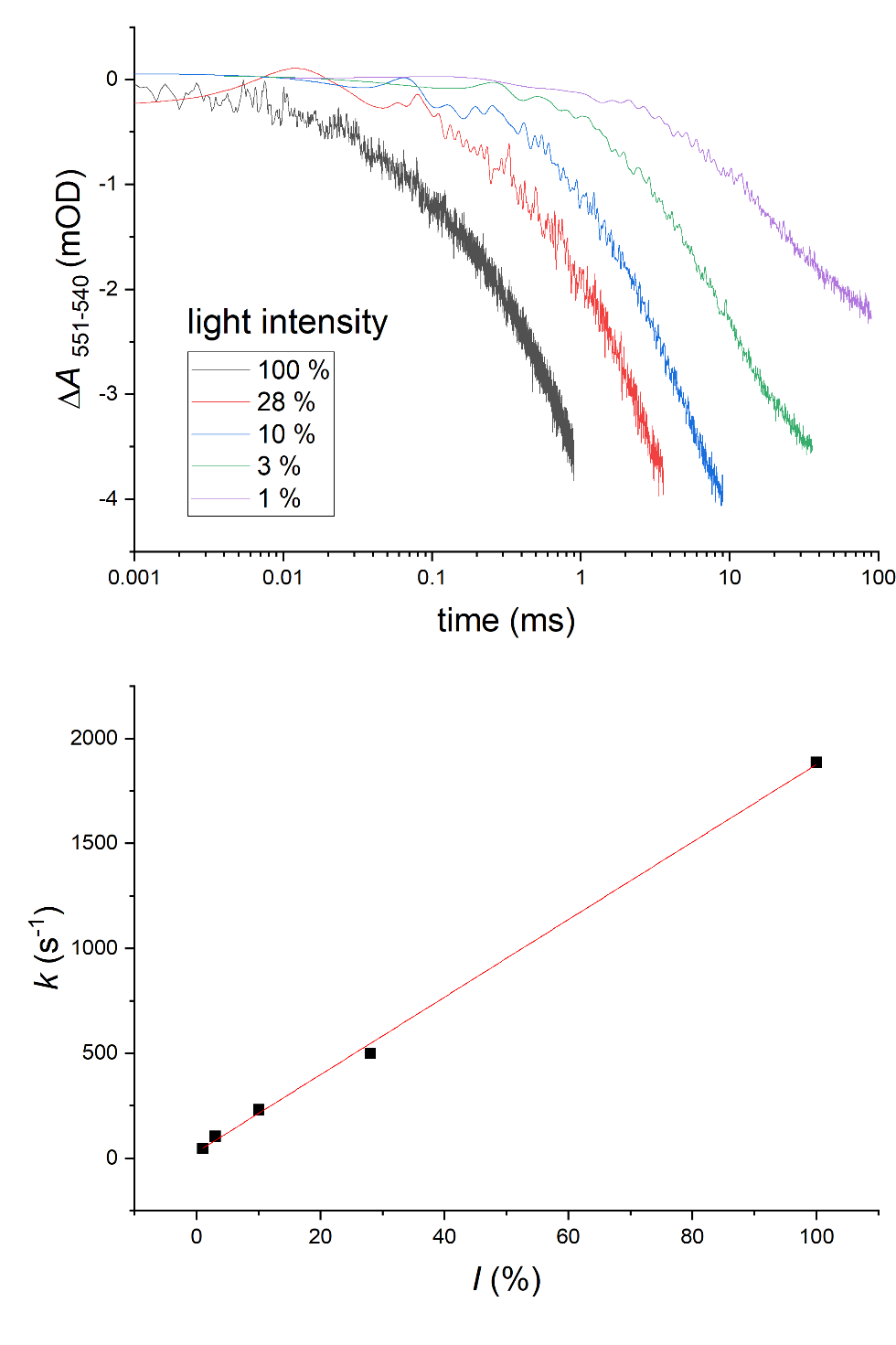


**Figure S2.** Dependence of the rate constants (*k*) of photooxidation of cytochrome *c* (Δ*A*_551-541_) on the intensity of the light excitation (*I*): proof of the photochemical nature of the cytochrome oxidation in intact cells of *Rvx. gelatinosus*. To get comparable results, the excitation light intensity of the laser diode was attenuated by neutral density filters (top) and the rate constants (slopes of the logarithm of cytochrome oxidation vs time) were plotted against the light intensity (bottom).


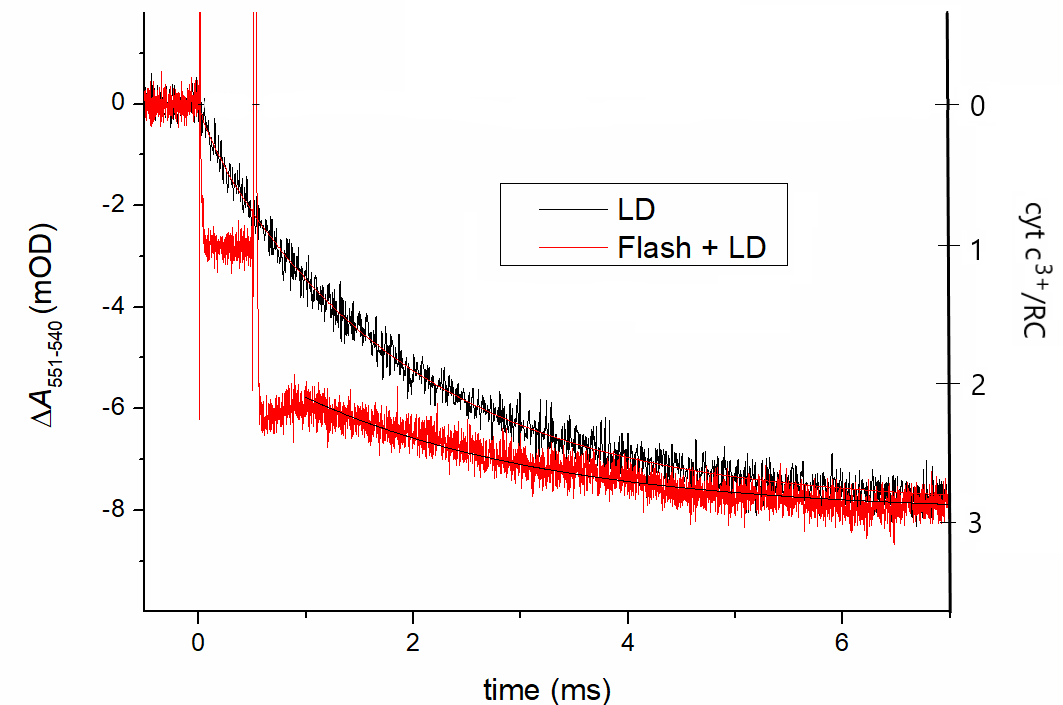


**Figure S3.** Kinetics and stoichiometry of photo-oxidation of cytochrome upon excitation by continuous laser diode (black) and by two saturating Xe flashes followed by laser illumination in intact cells of *Rvx. gelatinosus*. The vertical scales show change of optical density (OD) measured at 551 nm (vs. 540 nm) and the amount of oxidized cytochrome relative to that of RC (normalized to the first flash).


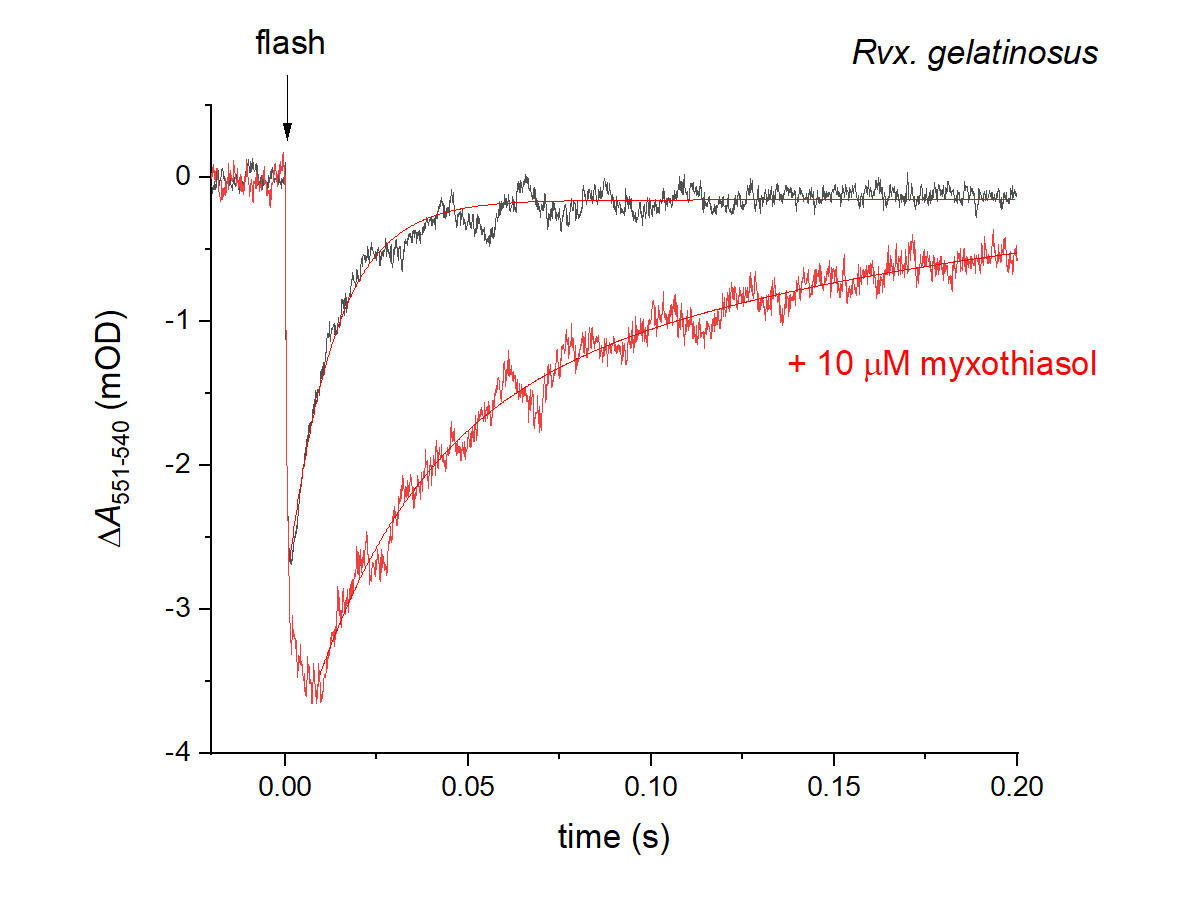


**Figure S4**. Effect of inhibitor of the cyt *bc*_1_ complex on the re-reduction kinetics of the flash-oxidized cytochrome in intact cells of *Rvx. gelatinosus*: with (red) and without (black) 10 μM myxothiasol. The re-reduction of the photo-oxidized cytochromes by the *bc*_1_ complex occurs on the 10 ms time scale (lifetime 13 ms) that is increased by an order of magnitude (lifetime 150 ms) by the cyt *bc*_1_ inhibitor.
